# Reconstruction of Endometrium from Human Endometrial Side Population Cell Lines

**DOI:** 10.1371/journal.pone.0021221

**Published:** 2011-06-21

**Authors:** Irene Cervelló, Aymara Mas, Claudia Gil-Sanchis, Laura Peris, Amparo Faus, Philippa T. K. Saunders, Hilary O. D. Critchley, Carlos Simón

**Affiliations:** 1 Fundación IVI-Instituto Universitario IVI, Universidad de Valencia, INCLIVA, Valencia, Spain; 2 MRC/University of Edinburgh Centre for Reproductive Health, Queen's Medical Research Institute, The University of Edinburgh, Edinburgh, Scotland, United Kingdom; 3 Valencian Node of the Spanish Stem Cell Bank, Prince Felipe Research Centre (CIPF), Unidad Mixta CIPF-UVEG, Valencia, Spain; University of Córdoba, Spain

## Abstract

Endometrial regeneration is mediated, at least in part, by the existence of a specialized somatic stem cell (SSC) population recently identified by several groups using the side population (SP) technique. We previously demonstrated that endometrial SP displays genotypic, phenotypic and the functional capability to develop human endometrium after subcutaneous injection in NOD-SCID mice. We have now established seven human endometrial SP (hESP) cell lines (ICE 1–7): four from the epithelial and three from the stromal fraction, respectively. SP cell lines were generated under hypoxic conditions based on their cloning efficiency ability, cultured for 12–15 passages (20 weeks) and cryopreserved. Cell lines displayed normal 46XX karyotype, intermediate telomerase activity pattern and expressed mRNAs encoding proteins that are considered characteristic of undifferentiated cells (Oct-4, GDF3, DNMT3B, Nanog, GABR3) and those of mesodermal origin (WT1, Cardiac Actin, Enolase, Globin, REN). Phenotype analysis corroborated their epithelial (CD9+) or stromal (vimentin+) cell origin and mesenchymal (CD90+, CD73+ and CD45−) attributes. Markers considered characteristic of ectoderm or endoderm were not detected. Cells did not express either estrogen receptor alpha (ERα) or progesterone receptor (PR). The hESP cell lines were able to differentiate *in vitro* into adipocytes and osteocytes, which confirmed their mesenchymal origin. Finally, we demonstrated their ability to generate human endometrium when transplanted beneath the renal capsule of NOD-SCID mice. These findings confirm that SP cells exhibit key features of human endometrial SSC and open up new possibilities for the understanding of gynecological disorders such as endometriosis or Asherman syndrome. Our cell lines can be a valuable model to investigate new targets for endometrium proliferation in endometriosis.

## Introduction

The existence of somatic stem cells (SSC) in the human and murine endometrium has been postulated and attributed to a particular subpopulation of mesenchymal origin located in the basal layer [1]–[16]. Nevertheless, the main limitation of these studies is the absence of specific markers that identify the endometrial SSC population.

The side population (SP) phenotype was first described in bone marrow where a somatic stem cell population was identified based on the ability of these cells to extrude the DNA binding dye, Hoechst 33342 [17] a phenomenon associated with the expression of an ATP binding cassette transported encoded by the breast cancer resistance protein gene (*BCRP1*/*ABCG2*) [18]. In general, the SP phenotype is thought to be a universal marker of somatic stem cells and has been used to isolate them from many adult tissues, such as the mammary gland, skin, myometrium, lung and dental pulp [19]–[23]. Accumulated evidence has suggested that the endometrial SP population could represent, at least in part, the SSC population in this particular tissue [24]–[27]. Functional proof of concept has been demonstrated by the ability of endometrial SP to reconstruct human endometrium after subcutaneous injection [26] or transplantation under the kidney capsule [27] in NOD-SCID mice. Nevertheless, the efficiency of this endometrium reconstruction is very low, as only two cases from the total of 24 injected animals [27] and one out of 50 [26] were successful.

To further explore our previous finding [26], the aim of the present study was to establish and characterize human endometrial SP cell lines from the epithelial and stromal compartments providing a model system for studies on human endometrial SSC.

## Methods

### Ethics statement

This study was approved by the Institutional Review Board and Ethics Committee of Instituto Universitario-IVI (Universidad de Valencia, Spain), and informed written consent was obtained from each patient prior to tissue collection. Procedures performed on animals were also approved by CIPF and Instituto Valenciano de Infertilidad (IVI) review boards (Animal ethical Committee 08/0065).

### Human tissue collection

Human endometrium from healthy women was obtained from endometrial biopsies using a Pipelle sampler (Genetics, Namont-Achel, Belgium) under sterile conditions.

### Epithelial and stromal separation

Endometrial biopsies were disaggregated by mechanical and enzymatic means in order to obtain single cell suspensions. Then, epithelial and stromal single cells were isolated from human endometrial tissue using established protocols [26], [28]. Cell suspensions were also treated with erythrocyte lysis solution (ammonium chloride, potassium bicarbonate, tetrasodium ethylene-diaminetetraacetic acid (EDTA)) to avoid red blood cell contamination by hypotonic shock, and evaluation of cell viability was performed with Propidium Iodide (PI; 5 µg/ml,Sigma-Aldrich, Spain).

### Flow cytometry and side population isolation

Epithelial and stromal cell suspensions were labeled with 10 µg/ml of Hoechst 33342 dye (Sigma-Aldrich, Spain) either alone or in combination with 50 µM verapamil (Sigma-Aldrich, Spain) in order to obtain Side Population cells, according to a previously described protocol [26]. Labeled cells were analyzed and sorted by a MoFlo® (Dako, Denmark, http://www.dako.com) jet-in-air high-speed sorter as described previously by Cervelló et al., 2010 [26]. The gates for cell sorting were defined to collect live cells with a low Hoechst 33342 fluorescence (SP fraction), as well as live cells with a high Hoechst 33342 fluorescence (NSP fraction).

### Cell culture and generation of cell lines

Cell lines generated were established in several steps. Briefly, the human endometrial SP cells from epithelial and stromal fractions isolated by flow cytometry were plated at clonal density (20–200 cells/cm2) and cultured in DMEM F-12 (Invitrogen, Spain) containing 10% of fetal bovine serum (FBS), glutamine 2 mM (Sigma-Aldrich, Spain), and antibiotic-antimycotic solution (Amphotericin B-Gentamicin). These cells were maintained at 37°C in hypoxic conditions (1–2% O_2_). Two weeks after seeding, the largest individual primary clones derived from single cells were selected, picked, and re-plated to generate secondary clones. After an additional two weeks in culture, secondary clones were picked and re-cloned to generate tertiary clones. At the fourth and following passages, cells reached 80–100% confluence. Serial cell culture continued in this manner through 12–15 passages.

### Immunophenotypic characterization

Once the putative epithelial and stromal somatic stem cell lines were established, a preliminary characterization was necessary to confirm purity, origin, and features of the generated cell lines. Analysis was assessed by the staining with typical antibodies: CD9 for epithelial cells; vimentin for stromal cells; CD90, CD73, and CD105 for mesenchymal stem cells; CD34 and CD45 for hematopoietic stem cells; CD31 for endothelial cells; Stro-1 for bone marrow stromal cells, and CD133 for endothelial progenitor cells (antibodies are detailed in supporting information Table S1). Immunophenotypic analysis was performed in a Cytomics FC500 flow cytometer (Beckman-Coulter, CA, USA).

### RNA isolation and reverse transcription

Total RNA extraction was performed by two methods depending on the desired outcome. For molecular characterization, RNA was extracted from cell cultures using the Mini RNA Isolation I Kit according to the manufacturer's protocol (Zymo, Irvine, CA 92614, U.S.A.). In addition, the TRIzol method was used in order to assess differentiation results (Life Technologies Inc., Gaithersburg, MD). In both cases, one µg of RNA was used for cDNA synthesis with the Reverse Transcription System (Clontech, Palo Alto, CA, USA).

### PCR for undifferentiated and differentiated markers

A semiquantitative PCR was performed for typical undifferentiated and differentiated markers in order to characterize the cell lines. The primers used are listed in the Table 1 (list of primers used for cell line characterization). *GAPDH* expression was used as a housekeeping gene for normalization.

**Table 1:** List of primers used for cell line characterization.

*Gene*	*Fw 5′-3′*	*Rv 5′-3′*	*Characterization*
*Oct-4*	*AAGAACATGTGTAAGCTGCGGCCC*	*GGAAAGGCTTCCCCCTCAGGGAAAGG*	*Undifferentiation*
Nanog	GATTTGTGGGCCTGAAGAAA	TGGGGTAGGTAGGTGCTGAG	*Undifferentiation*
GDF3	*CTTCACCCCAGAAGTTCCAA*	*GCAGGTTGAAGTGAACAGCA*	*Undifferentiation*
GABR3	CTTGACAATCGAGTGGCTGA	*CAACCGAAAGCTCAGTGACA*	*Undifferentiation*
DNMT3B	TTTGGCCACCTTCAATAAGC	*GGCAACATCTGAAGCCATTT*	*Undifferentiation*
WT1	TCCTTCATCAAACAGGAGCCGAGC	*CTGTAGGGCGTCCTCAGCAGCAAAG*	*Mesoderm origin*
Renin	AGTCGTCTTTGACACTGGTTCGTCC	*GGTAGAACCTGAGATGTAGGATGC*	*Mesoderm origin*
Cardiac Actin	TCTATGAGGGCTACGCTTTG	*CCTGACTGGAAGGTAGATGG*	*Mesoderm origin*
Enolase	TGACTTCAAGTCGCCTGATGATCCC	*TGCGTCCAGCAAAGATTGCCTTGTC*	*Mesoderm origin*
δ-Globin	ACCATGGTGCATCTGACTCCTGAGG	*ACTTGTGAGCCAAGGCATTAGCCAC*	*Mesoderm origin*
AMY	GCTGGGCTCAGTATTCCCCAAATAC	*GACGACAATCTCTGACCTGAGTAGC*	*Endoderm Origin*
NFH	TGAACACAGACGCTATGCGCTCAG	*CACCTTTATGTGAGTGGACACAGAG*	*Ectoderm origin*
GAPDH	TGAGCTGAACGGGAAGCTCA	*GTCTACATGGCAACTGTGAGGA*	*Housekeeping*

The molecular analyses of these genes were carried out in ICEp and ICEs cell lines. Human embryonic stem cell (hESC) line VAL-9 [29] was used as a positive control for undifferentiated markers and diverse tissues (heart, kidney, etc.) were used to test mesodermal origin. PCR was carried out using a BIOMETRA thermal cycler, with the following conditions 59°C and 30 cycles. Finally, the PCR products were run on a 2% agarose electrophoresis gel.

### Karyotype

In passage 8 to 9, the cells were treated with culture medium containing colchicine (KaryoMAX®Colcemid™ solution, Invitrogen) for 30 minutes and treated with hypotonic buffer 0.075 M KCl (Potassium Chloride, Invitrogen) during 15 minutes. Cell suspensions were fixed in 3∶1 methanol-acetic acid and air-dried. Finally cells were stained with 4′,6-diamidino-2-phenylindole (DAPI, Invitrogen) to performed cytogenetic analysis.

### Telomerase activity

Telomerase activity was analyzed using the TRAP_EZE_® Telomerase Detection Kit (Chemicon), and further staining was performed with SYBR® Green I (Molecular probes). Briefly, cultured cells (100,000 cells) were harvested, washed once in Ca^2+^/Mg^2+^-free PBS, and were immediately resuspended in lysis buffer. After treatment on ice and spinning at high speed, samples were subjected to a PCR reaction following the manufacturer's instructions. PCR products were run in polyacrylamide gel (BioRad) under non-denaturing conditions and amplified fragments were then stained with SYBR green for visualization in a transilluminator. Each experiment included the human embryonic stem cell line, VAL-9 as a positive control, and foreskin (somatic cell line) as a negative control.

### Estrogen and progesterone receptor expression

To investigate the expression of estrogen receptor alpha (ERα) and progesterone receptor (PR) in human endometrial SSC lines (ICEp and ICEs), cytospins were made from the cell suspensions and fixed in 90% acetone/10% methanol. Cytospins of the human breast adenocarcinoma MCF-7 (ATCC reference HTB22) and estrogen-treated human endometrial adenocarcinoma Ishikawa (ISH) (ECACC reference 99040201) cell lines served as positive controls for ERα and PR antibodies, respectively. Immunocytochemistry was performed as follows: endogenous peroxidase was blocked using a solution of 0.15% H_2_O_2_ (Fisher Scientific, Leicestershire, UK) in methanol before permeabilizing the cytospins in 0.2% IGEPAL, 1% BSA (both Sigma-Aldrich, Steinheim, Germany), and 10% normal goat serum (NGS) (Biosera, East Sussex, UK). Non-specific binding was blocked using 20% NGS/5% BSA before endogenous strepavidin and biotin were blocked per kit instructions (Vector Labs, Burlingame, CA). The mouse monoclonal primary antibodies ERα (Vector Labs, Burlingame, CA) and PR (Novocastra Laboratories, Newcastle upon Tyne, UK) were each diluted 1∶20 in blocking serum and applied to cytospins overnight at 4°C. The primary antibody was omitted on negative control slides. A biotinylated goat anti-mouse secondary antibody (1∶500 dilution, Vector Labs, Burlingame, CA) followed by HRP-conjugated strepavidin (1∶1000 dilution, Vector Labs, Burlingame, CA) were both applied for 30 minutes at room temp before addition of the substrate diaminobenzidine per kit instructions (Vector Labs, Burlingame, CA). The sections were counterstained in haematoxylin and mounted in pertex.

### *In vitro* differentiation

The ICEp and ICEs lines were cultured *in vitro* with adipogenic and osteogenic differentiation media. Normoxic conditions were used to maintain the cells under differentiation protocols (18–20% O_2_, 37°C, 5% CO_2_, 90% humidity). Both cell lines were treated with the adipogenic induction media: h-insulin, L-Glutamine, MCGS, Dexamethasone, Indomethacin, IBMX and Penicillin/streptomycin (Lonza, Barcelona, Spain), and osteogenic induction media: Dexamethasone, L-Glutamine, Ascorbate, MCGS, Penicillin/streptomycin and β-Glycerophosphate (Lonza), during two-three weeks according established manufacturer's protocols. Cells treated with the maintenance media were the negative control of the differentiation process. Afterward, cells were fixed and the adipogenic/osteogenic differentiation was detected by particular staining and specific gene expression. In the adipogenic differentiation procedure, Oil Red O staining and mRNA levels of specific genes, such as lipoprotein lipase, were used to detect the presence of lipid vacuoles [30]. For osteogenic differentiation, bone sialoprotein (BSP, MAB1061, Chemicon International) immunocytochemistry was performed and expression of osteoblast marker Osterix [31], [32] was measured at the mRNA level. RNA extraction and real-time PCR were performed to ensure the differentiation results. Our positive controls for Oil Red O and LPL expression were adipocyte cells cultured from tissue explants. Positive controls for bonesialoprotein expression and genes battery were osteocytes removed from bone explants. In all cases, negative controls were cultured endometrial cells.

### Animal Model: Xenotransplantation assays

All procedures involving animals in this study were approved by CIPF and Instituto Valenciano de Infertilidad (IVI) review board.

Female NOD-SCID mice (strain code 394; NOD. CB17- Prkdc^scid^/NCrCrl from Charles River Laboratories, Spain) were ovariectomized at 5–6 weeks and then used for xenotransplantation experiments. Subsequently, mice were anesthetized with sevofluorane inhalation followed by kidney externalization through a dorsal-horizontal incision for the cell injection. In order to guarantee the optimal technical procedure, we performed this procedure on two mice treated with the complete endometrial fraction, as Masuda described in PNAS 2007 [33].

In our experimental approach, the single-cell suspensions (200,000 to 1,000.000 cells) from ICEp and ICEs were resuspended in 30 µl of medium (DMEMF-12, Sigma-Aldrich, Spain) and injected under the kidney capsule. Negative controls were injected using only medium. During transplantation, estradiol pellets (SE121, 17β-estradiol 0.18 mg/60 days; Innovative Research of America) were also implanted subcutaneously at the neck. Moreover, some mice treated with estradiol pellets were injected subcutaneously every day with 1 mg of progesterone (P (P4), Dr. Carreras, Hospital14, Barcelona, Spain) for two weeks within a 3-week interval. After this period, mice were subjected to a second cycle of daily P4 injections for 2 weeks until they were sacrificed. Animals were injected with ICEp, ICEs and ICEp+ICEs. Throughout this assay, xenotransplanted mice were maintained in specified pathogen free (SPF) facilities and fed “ad libitum” for 60 days after the injection. After that, mice were nephrectomized according to the experimental protocol.

## Results

### SP isolation and generation of the endometrial SP epithelial and stromal cell lines

We first isolated the SP population from both fractions of the human endometrium as described in our previous work [26]. SP represents 1.68±0.27% of the total living cell population in the epithelial fraction and 0.39±0.06% of the stromal fraction.

The establishment of SP cell lines required the following steps. SP cells isolated from both endometrial fractions were cultured at clonal densities (20–200 cells/cm^2^) under hypoxic conditions for 2 weeks. The largest colonies, described as putative SSC [28], were re-plated at least three times to purify the cell population obtained. Subcultures were performed every 7–9 days and the generated cell lines required a change of medium after 3–4 days. After 12 to 15 passages (7 to 9 days per passage) a total of seven human endometrial SP cell lines were obtained (ICE1-7) and cryopreserved. Four lines were from epithelium and 3 lines from stroma. Two lines of epithelial origin (ICEp) corresponding to ICE1 and ICE6, and two from the stromal compartment (ICEs) corresponding to ICE2 and ICE7 were selected for full characterization.

### Phenotype of endometrial SP lines

**Figure 1:**
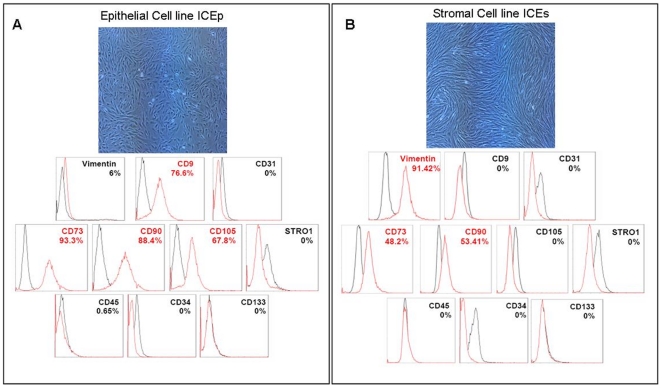
Morphology and phenotype of endometrial somatic stem cell lines (ICE lines). A. Epithelial cell line (ICEp). Upper panel shows aspect of cell growth in hypoxic conditions with typical epithelial features as polygonal/cuboidal shape. Lower panel, expression patterns related flow cytometric analysis confirms positive expression (highlighted in red) of ICEp for epithelial CD9 (76.6%) and for mesenchymal stem cell markers like CD73 (93.3%), CD90 (88.4%) and CD105 (67.8%). Negative expression in ICEp was distinguished for stromal origin (Vimentin), hematopoietic stem cell markers (CD45 and CD34), endothelial cells (CD31), bone marrow stromal (stro-1) and endothelial progenitors (CD133). B. Stromal cell line (ICEs). Upper panel shows the confluence cell culture with fibroblast-like appearance in hypoxic conditions. Lower panel, expression patterns related flow cytometric analysis confirms positive expression (highlighted in red) of ICEs for stromal Vimentin (91.4%) and for mesenchymal stem cell markers like CD73 (48.2%) and CD90 (53.4%). Negative expression in ICEs was distinguished for epithelial origin (CD9), mesenchymal stem cell marker (CD105), hematopoietic stem cell markers (CD45 and CD34), endothelial cells (CD31), bone marrow stromal (stro-1) and endothelial progenitors (CD133). In all the cases mouse FITC-labeled IgG1 (Millipore), FITC-labeled IgG2b (Chemicon), APC-labeled IgG1 (Milteny Biotec), Alexa647-labeled IgM (Biolegend), and PE-labeled IgG1(Abcam) were used as isotypic controls (black) for staining of endometrial somatic stem cell lines (ICEp and ICEs).

ICEp cell lines displayed typical epithelial, round morphology (Figure 1A), whereas ICEs cells presented with a typical fibroblast-like appearance (Figure 1C). Phenotypic analyses by flow cytometry demonstrate that ICEp contained 76.6% CD9 positive cells and ICEs contained 91.4% vimentin positive cells. In all cases mesenchymal commitment was demonstrated by the expression of CD90, CD105, and CD73. In the case of ICEp, the percentages obtained were 88.4% for CD90, 67.8% for CD105, and 93.3% for CD73. For ICEs lines expression was 53.4% for CD90, 48.2% for CD73, with no detection of CD105. Moreover, we corroborated the absence of typical markers for hematopoietic (CD45 and CD34), endothelial (CD31), bone marrow stromal (stro-1), and endothelial progenitors (CD133) in all the cell lines analyzed (Figure 1B and 1D).

### Karyotype, molecular characterization and telomerase activity

**Figure 2:**
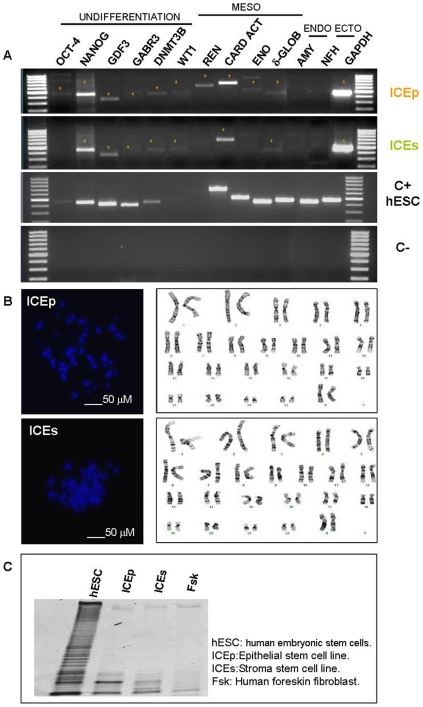
Molecular and cytogenetic characterization of ICE lines. A. Results from PCR assay demonstrated the expression of undifferentiated genes in both cell lines (highlighted in figure by asterisk). In addition the absence of typical endoderm and ectoderm markers suggest their commitment to mesenchymal lineage throughout the presence of mesoderm genes. To assess the integrity of the samples analysed GAPDH gene expression was performed. ICEp: Epithelial somatic stem cell line; ICEs: Stromal somatic stem cell line; C+: hESC, human embryonic stem cell line (VAL-9) and C−: water. B. Normal karyotypes 46XX were obtained in both cases. C. Telomerase activity associated with length of telomeres was performed in order to known the undifferentiated status of the cell lines. Telomerase activity of positive control, VAL, showed a ladder of amplification products with six base increments starting at 50 nucleotides. An intermediate telomerase pattern was observed in both cases in comparison with hESC (VAL-9) and differentiated cell line (Fsk).

After the establishment of putative endometrial somatic stem cell (hESC) lines, a preliminary molecular characterization was necessary to confirm their lineage, their cytogenetic integrity, and undifferentiated status. The molecular profiling of ICEp revealed the expression of genes characteristic of undifferentiated cells, such as *Oct-4 (Octamer-binding transcription factor 4), NANOG, GDF3 (Growth differentiation factor-3), GABR3, DNMT3B (DNA (cytosine-5-)-methyltransferase 3 beta)*, as well as those associated with mesodermal commitment: *WT1 (Wilms tumor protein), REN (Renin), Cardiac actin, ENO (Enolase)* and *δ-GLOB (gamma globulin)* (Figure 2A). ICEs also expressed a similar panel of genes including *NANOG*, *GDF3*, *GABR3* and *DNMT3B* as well as the mesodermal genes *WT1*, *Cardiac actin* and *δ-GLOB* (Figure 2A). These cell lines did not express either endodermal or ectodermal genes, such as *AMY (Amylase)* and *NFH (Neurofilament heavy chain)*. Importantly a normal karyotype corresponding to 46XX was confirmed in ICEp and ICEs at passages 8 to 9 (Figure 2B). The pattern of telomerase activity in these lines indicated it was intermediate between the human embryonic stem cell line (hESC) VAL-9 [29] and human foreskin fibroblast (Figure 2C).

### Immunoexpression of estrogen receptor alpha (ERα) and progesterone receptor (PR)

**Figure 3:**
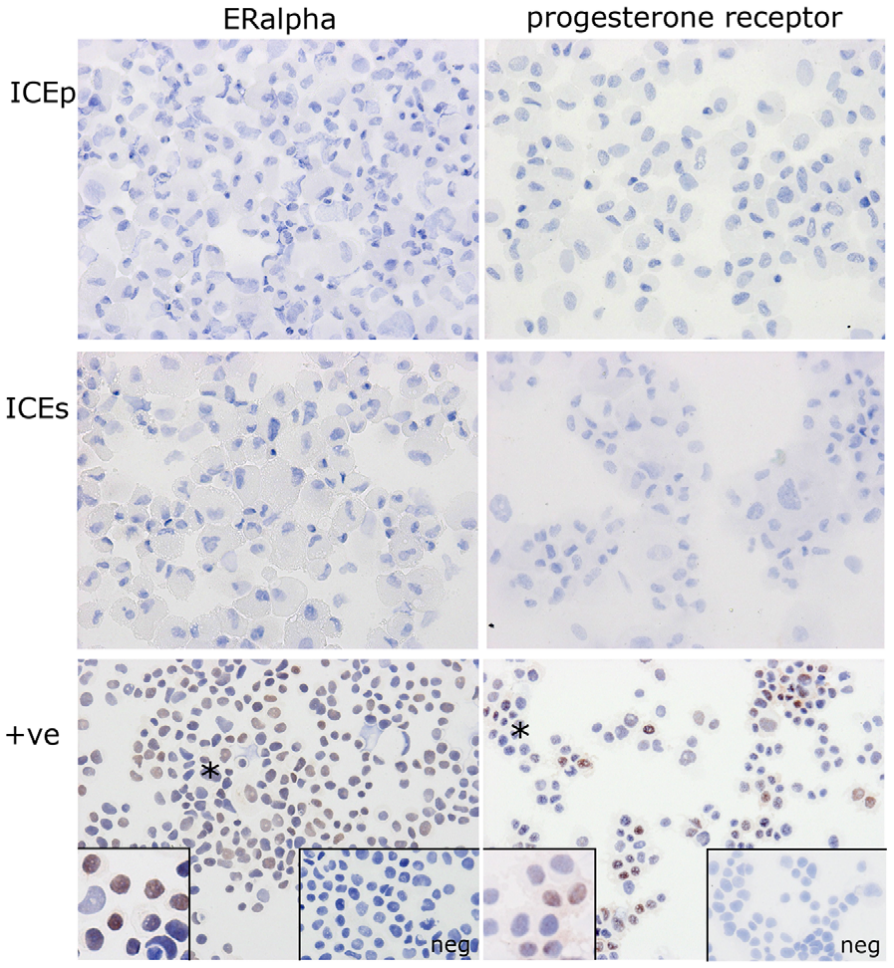
Immunostaining for ERalpha and progesterone receptor on cytospins of ICE and control cell lines. Immunopositive staining for ERalpha was detected in the nuclei of HTB222 cells (lower left panel, insets high magnification and negative control). Immunopositive staining for progesterone receptor was detected in nuclei of Ishikawa cells grown in the presence of E2 (lower left panel, insets high magnification and negative control). In both the positive contol (+ve) cell lines a proportion of the cell nuclei were immunonegative (*). No immunopositive cell nuclei were detected in any of the ICEp or ICEs cell lines tested. All ICE cell cytospins were stained in parallel with the appropriate positive control cell line using identical conditions.

Immunocytochemical studies were performed to determine whether the SP cell lines generated expressed the sex steroid receptors, ERα and PR. ERα was not detected in the cellular suspensions analyzed from either ICEp or ICEs (Figure 3A). In contrast, strong expression of ERα was observed in HTB22, a commercial cell line derived from adenocarcinoma of human mammary gland that served as a positive control. Furthermore, PR was not detected in ICEp or ICEs (Figure 3B) although Ishikawa cells treated with estradiol (E_2_) were immunopositive.

### Adipogenic and osteogenic differentiation of SP cell lines

The ability to differentiate into different cell types from a specific lineage is one of the features of SSC cells. We examined the *in vitro* potential of the SP cell lines ICEp and ICEs to undergo osteogenic and adipogenic differentiation. At passage 6, both cell lines were further cultured in the presence of adipogenic or osteogenic induction media (see Materials and Methods) in normoxic conditions.

**Figure 4:**
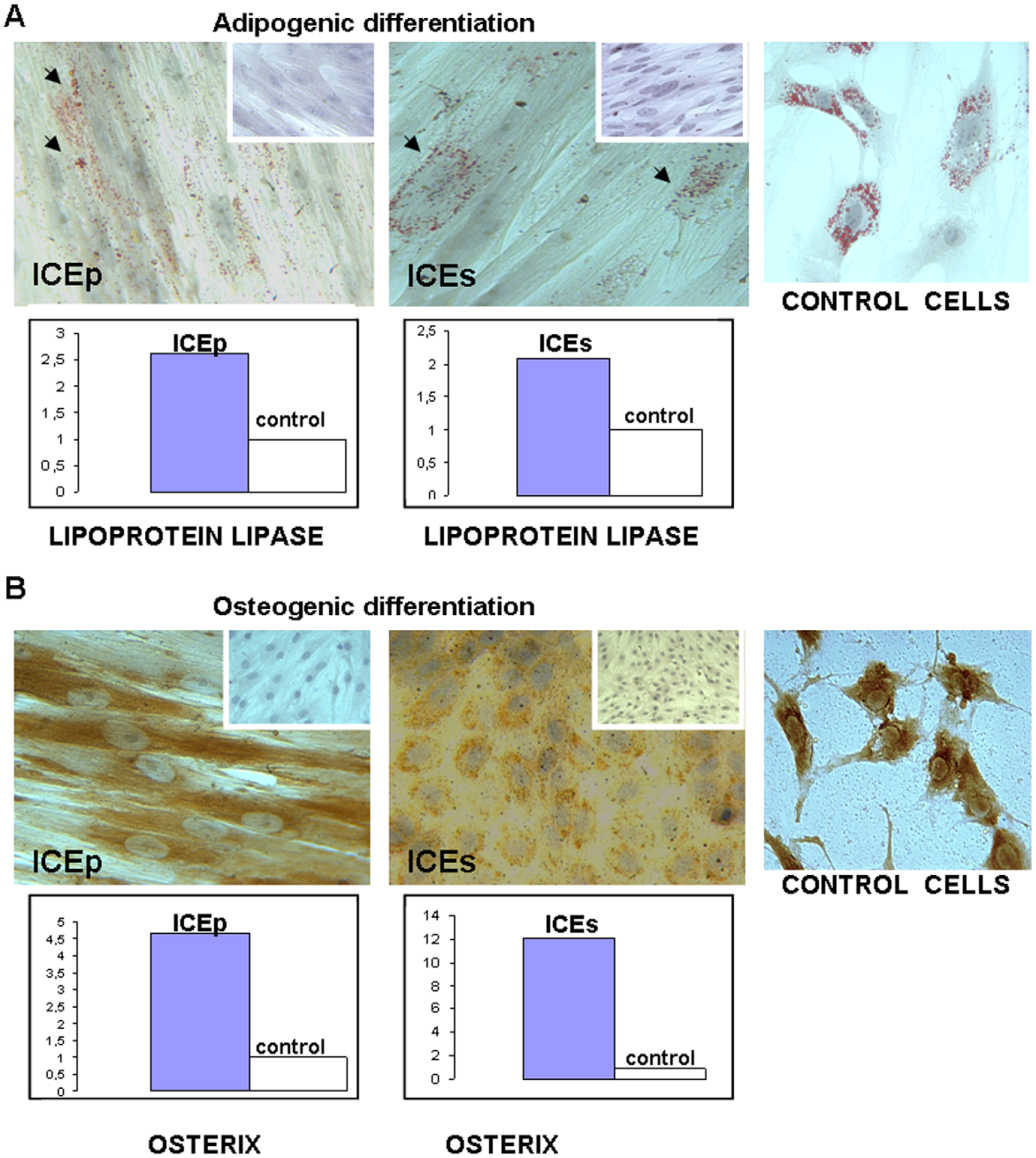
*In vitro* differentiation of ICE lines into mesenchymal lineages. A. Upper panel, adipogenic differentiation assay was visualised by the presence of Oil Red O staining in lipids vacuoles. Cells non-treated with differentiation media were included as control, being negative for Oil Red staining. Adipocytes from culture explant were included as positive control. Lower box indicated over-expression of lipoprotein lipase at mRNA levels in induced differentiated cells. B. Upper panel, osteogenic differentiation process was detected by the reactivity against bone sialoprotein. Cells non-treated with differentiation media were included as control being negative for bone sialoprotein expression. Osteocytes from culture explant were included as a positive control. Lower box shows over-expression of osterix mRNA in induced differentiated cells.

Adipogenic differentiation was assessed by the accumulation of lipid droplets demonstrated by Oil Red staining in the cytoplasm of differentiated cells. Positive staining for lipid vacuoles was confirmed in the cytoplasm of all cells differentiated from ICEp and ICEs. Moreover, at the molecular level adipogenic transformation was demonstrated by the increased expression of lipoprotein lipase (LPL) [30] (Figure 4A). Osteogenic differentiation was investigated by an immunocytochemistry assay using the specific biochemical marker of mineralized tissues, bonesialoprotein (BSP). It was also confirmed at the molecular levels by the increased expression of osterix in both cell lines (Figure 4B) [31], [32]. Therefore, similar to the results obtained with SP cells [26], ICEp and ICEs were able to differentiate *in vitro* into adipocytes and osteocytes using standard protocols.

### Reconstruction of human endometrial-like tissue from SP cell lines in NOD-SCID mice

**Figure 5:**
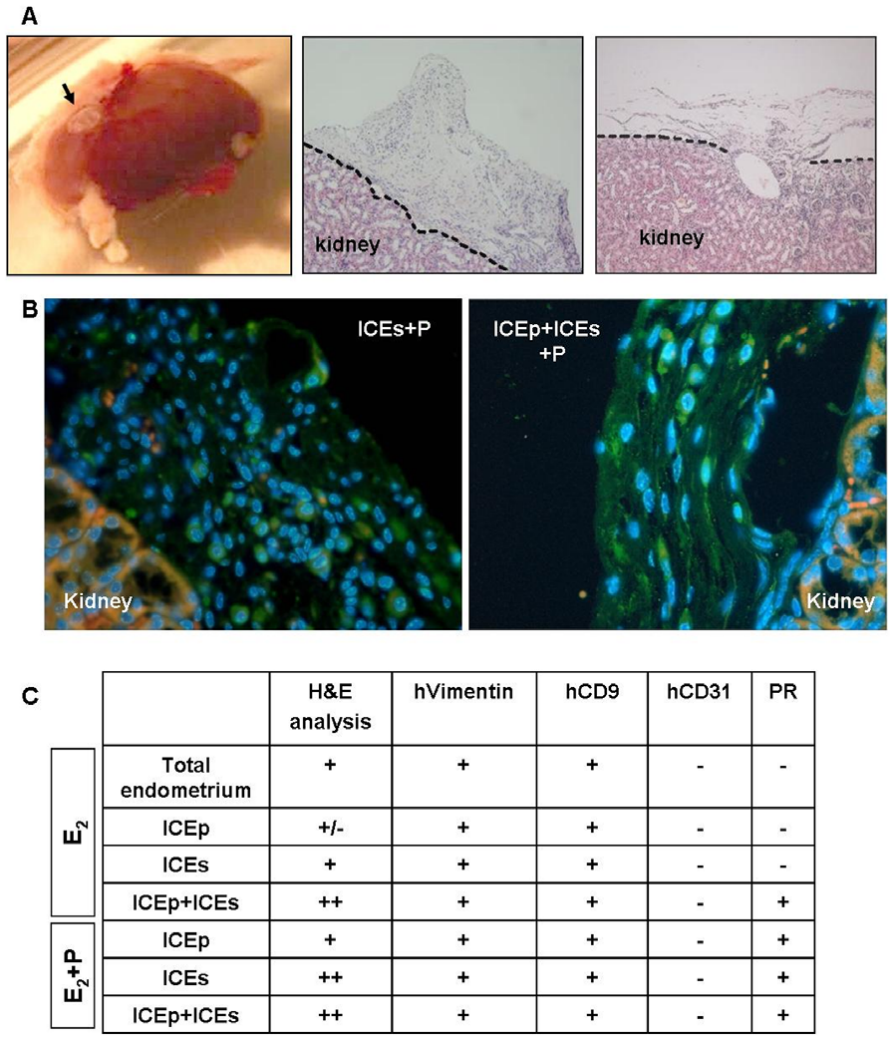
Reconstruction of human endometrial-like tissue from ICE lines. A. Macroscopic and microscopic visualisation of the transplanted site (arrow). H&E staining to assess the presence of endometrial reconstruction in the murine kidney after 60 days of xenotransplantation. B. Pictures showing some results for human vimentin protein expression (green fluorescence signal) in mice treated with P4 and injected with stromal (ICEs) and epithelial+stromal (ICEp+ICEs) cell lines. Nuclei stained with DAPI (blue fluorescence signal) and autofluorescence of kidney cells (red fluorescence signal) are shown. C. Table summarizing the results of the samples analysed. In the first column H&E results concerning visual analysis are assigned as + (reconstructed endometrial tissue), ++ (high appearance of endometrial-like tissue) and +/− (poor observation of reconstructed tissue) in all the mice injected with human cells. Serial markers for immunohistochemistry assays (hVimentin, hCD9, hCD31 and PR) were performed in a subset of mice injected with total endometrium, ICEp, ICEs, ICEp+ICEs, ICEp+P4, ICEs+P4 and ICEp+ICEs+P4. Note positive expression is indicated as + and negative expression as −.

Single-cell suspensions composed by 200,000 to 1,000,000 cells obtained from ICEp, ICEs or ICEp+ICEs at passage 6 were injected under the kidney capsule of immunosuppressed mice treated with E_2_ or E_2_+P (see Materials and Methods). Mice treated with single cell suspensions (500,000 cells) of total endometrium were used as the positive control of the experimental procedure [33]. In all cases, the injection of ICEp, ICEs or ICEp+ICEs and treatment of the host with E_2_, or E_2_+P resulted in the reconstruction of endometrial-like tissue in the kidney capsule (Figure 5A and 5C). The endometrial reconstruction was uncertain in only one case of ICEp exposed to E_2_ (Figure 5C).

**Figure 6:**
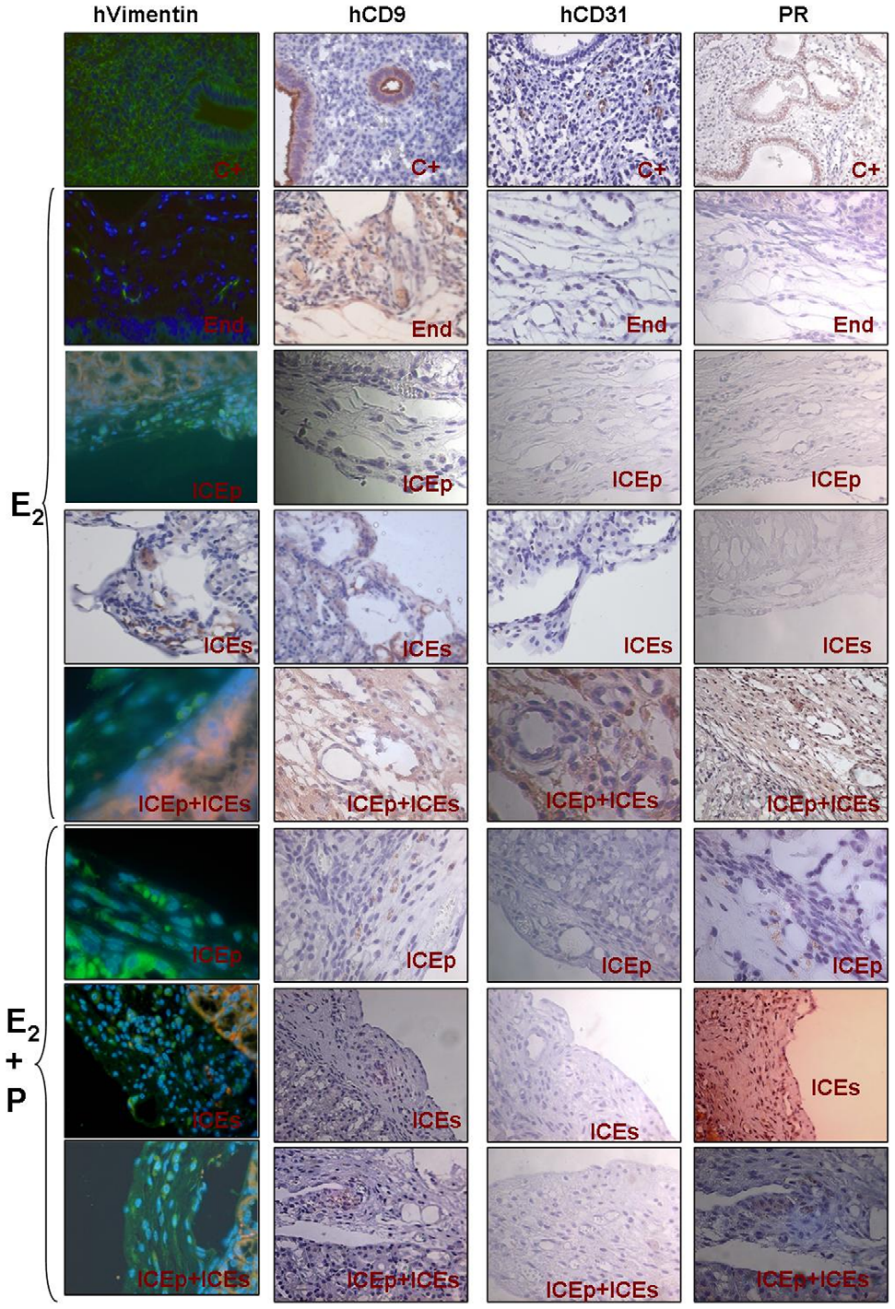
Immunohistochemical analysis of endometrial xenografts from mice. Panel summarizing the immunohistochemical analysis for hvimentin, hCD9, hCD31 and PR in endometrial-like tissues obtained after the injection of total endometrial cell suspensions (End = positive control of the cellular injection procedure) treated with E_2_, putative epithelial somatic stem cell lines from human endometrium (ICEp) treated with E_2_ and E_2_+P, putative stromal somatic stem cell lines from human endometrium (ICEs) treated with E_2_ and E_2_+P and ICEp+ICEs treated with E_2_ and E_2_+P. Human endometrial tissue sections were used as positive controls for all the antibodies described above (upper section of the panel).

To further assess the human origin of the reconstructed endometrial tissues, we performed immunohistochemical analysis of the kidney capsules removed from all cell lines as well as cell lines with hormonal treatment using human vimentin Figure 5B), human CD9, human progesterone receptor, and human CD31 with proper controls (see Figure 6). Results are presented in Figure 6 and summarized in Figure 5C. The results demonstrated that endometrial SP lines were capable of reconstructing total human endometrium determined by the presence of human vimentin and CD9 positive cells in the renal capsule. We further confirmed the expression of human progesterone receptor (PR) in all the xenografts obtained from animals treated with E_2_+P. Interestingly, in the endometrial-like tissue obtained in animals treated with E_2_ alone after the injection of cells from ICEp+ICEs the expression of PR was also detected. As expected, human endothelial marker hCD31 was not detected in endometrial-like tissue suggesting that the neovascularization derived from host.

## Discussion

The results of this study extend and confirm our previous finding suggesting that endometrial SP cells from both the stromal and epithelial compartment are putative human endometrial stem cells [26]. Here, we demonstrated that human endometrial SP (hESP) lines expressed markers characteristic of undifferentiated cells together with those of typical of cells committed to a mesodermal lineage. Notably these cells were able to differentiate into both adipocytes and osteocytes *in vitro*, and to give rise to endometrial-like tissue *in vivo*. We have demonstrated that hESP cells do not express ERα and PR and thus will be unresponsive to sex steroid action mediated via these two steroid receptors.

Due to the absence of specific markers for endometrial stem cells, we have used a two-step process for derivation of human endometrial SP cell lines. The first process is based on the Side Population (SP) method that relies on the capability of cells to extrude the DNA binding dye Hoechst 33342 via the ATP-binding cassette [17], [18]. This method has been used for the identification of putative SSC in skin [20], myometrium [21], lung [22], dental pulp [23] and endometrium [24]–[27]. Utilisation of a cloning efficiency method [6] under hypoxic conditions [26] has been undertaken to further select the subpopulation that gives rise to hESP lines. The hypoxic environment has been considered essential to maintain SP cells derived from bone marrow [34] and to enhance the proliferation of somatic stem cells [35], [36].

Using these selection criteria four hESP lines were established from epithelial compartment (ICEp lines 1-3-5-6) and three from the stromal compartment (ICEs, lines 2-4-7). Two ICEp and two ICEs were selected for full characterization and were shown to maintain a stable 46XX karyotype. Epithelial (CD9) and stromal (vimentin) cell markers confirmed their respective epithelial or stromal origin, and the additional demonstration of homogenous expression of WT1, cardiac actin, and δ-GLOB further suggested their mesenchymal origin [10], [26].

The coexistence of undifferentiated markers with those of mesodermal origin in the absence of ectoderm or endoderm differentiation genes have been identified in other cell types, such as adipose-derived stem cells [37], [38] and adult bone marrow-derived stem cells [39]. Our hESP cell lines, like other adult-derived stem cells described previously, not only co-expressed mesenchymal and differentiated markers at protein and molecular levels, but also demonstrated the capability to differentiate into mesodermal specific lineages such adipocytes and osteocytes when exposed to appropriate conditions.

Estrogen regulates endometrial cell survival, viability and mitogenic effects via ERα, the predominant endometrial estrogen receptor [40]. ERβ is expressed in multiple cell populations throughout the human endometrium [41] and studies in mice suggest it may have a negative impact on Erα mediated responses [42]. Mouse PR knockout studies indicate that the anti-proliferative effect of progesterone on endometrial epithelium is mediated by the PR-A isoform [43]. ICEp and ICEs did not express ERα or PR. This finding is in agreement with a report that ERα was not expressed in putative endometrial stem/progenitor cells identified using the label retaining cell (LRC) mouse model in which slow-cycling (putative stem cells) were identified by injection of bromodeoxyuridine [1]. In this study Chan and Gargett immunolocalised ERα to the nuclei of differentiated epithelial and stromal cells but found that epithelial LRC were ERα negative although a small population (16%) of stromal LRC did express ERα. A recent study has reported that, at least in mice, estrogen-induced proliferation of uterine epithelium is not mediated by expression of ERα [44] a finding in agreement with earlier studies using tissue recombinants that reported estrogen regulates endometrial epithelial proliferation through paracrine signaling involving ERα positive stromal cells [45]. Notably in both primates and mouse models during endometrial breakdown and early repair ERα is expressed in stromal but not in epithelial cells supporting the notion that the stromal compartment plays the key role in the orchestration of normal endometrial reconstruction in response to estrogen [46], [47].

Although ERα and PR were not detected in the hESP lines when these cells were injected into immunocompromised mice the stromal cells were able respond to exogenous E_2_ and P and the human epithelial cell layer formed in the reconstructed endometrium expressed PR (Figure 5). Therefore, we hypothesize that signals coming from the stem cell niche may maintain the SSC population in a hormonally naïve state and that they become responsive to steroids after differentiating into transient amplifying cells.

We have also shown that hESP cell lines retain the capability to differentiate in vitro into different mesodermal lineages, including adipocytes and osteocytes [26]. The key achievement of the current study was our ability to demonstrate that, when injected into the renal capsule of immunodeficient mice (NOD-SCID), hESP lines consistently generated endometrial human tissue. Notably, we have injected ICEp or ICEs lines both separately or in combination (ICEp+ICEs) into animals that were subsequently treated with E_2_ alone or E_2_+P. All cases (except one) this regimen resulted in the reconstruction of endometrial-like tissue in the kidney capsule (Fig. 5A, 5B and 6) that was also further characterized for the expression of human vimentin, hCD9, hCD31 and PR (Figure 5C and 6) resulting in the following findings. First, human endometrium could be regenerated from hESP lines obtained from the epithelial (ICEp) as well as from the stromal (ICEs) compartments. Second, the sequential treatment with E_2_+P was superior in terms of the amount of reconstructed endometrium produced. Finally, the vasculature of the newly formed endometrium was derived from the host since hCD31 was not detected in any of the xenografts analyzed.

In conclusion, we demonstrate that *in vitro* differentiation to adipocytes and osteocytes, as well as *in vivo* formation of endometrial-like tissue, can be obtained after renal capsule injection of cells from hESP lines that are chromosomally normal, ERα and PR negative, and committed to a mesoderm lineage. Therefore, we have demonstrated that hESP cell lines display similar phenotypic, molecular signatures, *in vitro* and *in vivo* differentiation capabilities as “primary” endometrial SP cells [26], creating a reliable in vitro model to test relevant targets for endometrial physiology and pathology.

The limitations however of the present study are that the hESP lines created have a limited number of passages, usually the total culture period is about 20 weeks, after which they cease to proliferate effectively. The next step is to further refine the model focusing on specific markers and mechanisms.

## Supporting Information

Table S1 List of antibodies used in the flow cytometric analysis.
(TIF)
