# Supplementary figures and images for: Reconstruction of Endometrium from Human Endometrial Side Population Cell Lines
Source: PLoS One. 2011 Jun 21;6(6):e21221. doi: 10.1371/journal.pone.0021221 (PMC3119688; doi:10.1371/journal.pone.0021221)

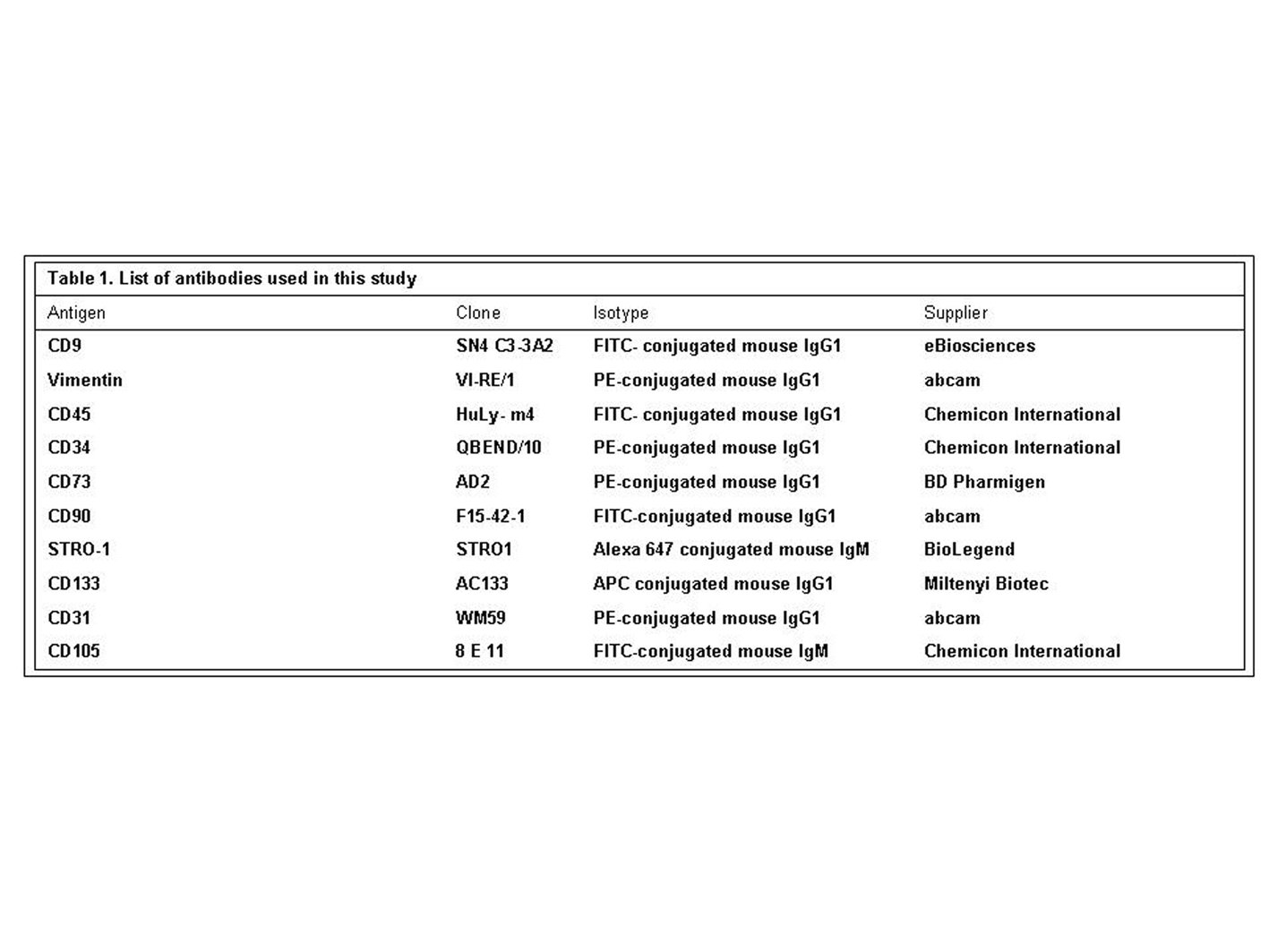

Supplement: Table S1 — List of antibodies used in the flow cytometric analysis. (TIF) [file pone.0021221.s001.tif]
